# A Socio-Technical Approach to Preventing, Mitigating, and Recovering from Ransomware Attacks

**DOI:** 10.4338/ACI-2016-04-SOA-0064

**Published:** 2016-06-29

**Authors:** Dean F. Sittig, Hardeep Singh

**Affiliations:** 1 University of Texas Health Science Center at Houston, School of Biomedical Informatics and UT-Memorial Hermann Center for Health Care Quality and Safety, Houston, Texas; 2 Houston Veterans Affairs Center for Innovations in Quality, Effectiveness and Safety, Michael E. DeBakey Veterans Affairs Medical Center, Houston, Texas; 3 Section of Health Services Research, Department of Medicine, Baylor College of Medicine, Houston, Texas

**Keywords:** Health information technology, electronic health record, socio-technical, cybersecurity, ransomware

## Abstract

Recently there have been several high-profile ransomware attacks involving hospitals around the world. Ransomware is intended to damage or disable a user’s computer unless the user makes a payment. Once the attack has been launched, users have three options: 1) try to restore their data from backup; 2) pay the ransom; or 3) lose their data. In this manuscript, we discuss a socio-technical approach to address ransomware and outline four overarching steps that organizations can undertake to secure an electronic health record (EHR) system and the underlying computing infrastructure. First, health IT professionals need to ensure adequate system protection by correctly installing and configuring computers and networks that connect them. Next, the health care organizations need to ensure more reliable system defense by implementing user-focused strategies, including simulation and training on correct and complete use of computers and network applications. Concomitantly, the organization needs to monitor computer and application use continuously in an effort to detect suspicious activities and identify and address security problems before they cause harm. Finally, organizations need to respond adequately to and recover quickly from ransomware attacks and take actions to prevent them in future. We also elaborate on recommendations from other authoritative sources, including the National Institute of Standards and Technology (NIST). Similar to approaches to address other complex socio-technical health IT challenges, the responsibility of preventing, mitigating, and recovering from these attacks is shared between health IT professionals and end-users.

## Introduction

Rapid adoption of electronic health records (EHRs) has fundamentally changed the way health care organizations and clinicians care for patients, manage the hospital, account for health care quality, and bill for their services. Recently there have been several high-profile ransomware attacks involving hospitals [1–6]. Furthermore, a recent survey of 61 chief information officers, chief information security officers, and other IT director-level respondents conducted by HIMSS Analytics (Chicago, IL) found that more than half of them had been targets of ransomware attacks in the previous 12 months [7]. Most of these organizations either a) fended off the attacks through intelligent use of network and user activity surveillance systems, b) were able to restore their critical systems from backups, or c) quietly paid the ransom. Reports of these events are generally leaked to the news media only after hospital operations are compromised for an extended period of time. In the absence of a centralized learning system for these events [8], it is not possible to decipher specific details of what happened, how it was initiated, who was responsible, and how it was resolved.

While specific details of how ransomware attacks begin are not well known, they often start when a user is tricked into clicking a link or opening an attachment of a malicious email message. Software that is intended to damage or disable the computer is then downloaded to the user’s computer, and it quickly encrypts all of the data on that machine and possibly reaches out over the network to encrypt data on other machines as well, thus rendering all data inaccessible [9]. The user is then presented a message stating that all the files have been encrypted, and if they do not pay a ransom within a short period of time, the files will be destroyed. Once the attack has been launched, users have three basic options: 1) try to restore their data from a backup; 2) pay the ransom; or 3) lose their data. The goal of this paper is to provide recommendations to health care organizations (HCOs) on how to prevent and mitigate these malicious events. We use a socio-technical approach to address ransomware and outline four overarching steps that organizations can undertake to secure an EHR and the underlying computing infrastructure.

## Origin of Ransomware

While ransomware in hospitals seems to be much discussed these days, the concept dates back to the distribution of the “AIDS Trojan virus” via floppy disk through surface mail back in 1989 [10]. Over fifteen years of internet revolution passed before the next instance of ransomware (GPCoder), which was delivered via e-mail in 2005 [11, 12]. The means of distribution of these ransomware programs has since grown to include malicious advertisements; USB drives; macros embedded in documents, spreadsheets, and presentations; archived files; batch or command files; and executables. The means of paying the ransom has also evolved from sending checks to off-shore bank accounts to paying via PayPal, from requiring users to purchase cash cards from certain websites, to paying with bitcoins. With increasing electronic data, malicious programs that encrypt key files and demand payment for the decryption key must now be taken seriously [13]. Moreover, health care has become more electronic than ever, making it attractive to hackers.

## Conceptual Approach to Addressing Health IT Ransomware

As with most health information technology-related (HIT) issues, preventing a ransomware attack is a complex socio-technical problem [14]. For example, ransomware attacks often rely on some form of “social engineering,” or the psychological manipulation of people in an attempt to gain their trust and lead them to divulge confidential information, along with a sophisticated encryption algorithm (i.e., technical part of problem). Solving these types of socio-technical problems is a shared task between those responsible for configuring, maintaining, and operating the organization’s HIT infrastructure as well as the users of this infrastructure. While preventing all ransomware attacks is not possible, there are a number of steps HCOs can take to reduce their risk as well as mitigate potential harm.

Based on previously developed health IT-related conceptual frameworks and the National Institute of Standards and Technology (NIST) Framework for Improving Critical Infrastructure Cybersecurity [15], we outline four overarching socio-technical steps to secure an EHR system and the underlying computing infrastructure [16, 17]. First, health IT professionals need to ensure adequate system protection by correctly installing and configuring computers and networks that connect them. Next, HCOs need to ensure more reliable system defense by implementing user-focused strategies, including simulation and training on correct and complete use of computers and network applications. Concomitantly, the organization needs to monitor computer and application use continuously in an effort to detect suspicious activities and identify and address security problems before they cause harm. Finally, organizations need to respond adequately to and recover quickly from a ransomware attack and take actions to prevent them in future.

In the sections below, we outline a comprehensive, multi-faceted socio-technical approach to preventing and mitigating these attacks. The detailed recommendations follow Sittig and Singh’s eight dimensional socio-technical model and are summarized in ► Table 1. These recommendations address all five functions of the NIST Cybersecurity Framework Core—Identify, Protect, Detect, Respond, Recover, thus providing HCOs with an operational strategy for management of ransomware risk. While some of the recommendations we suggest might be common-sense and obvious, often these mishaps occur from failing to adhere to the most basic recommendations. For example, the most likely cause of the attack on MedStar was “an improperly installed JBoss server” that “appears to have used the default settings leaving access to the server’s management interface open to the Internet” [18]. Even after the attack, many institutions remain vulnerable, as shown by an Internet scan revealing that 2.1 million systems still remain vulnerable to the same JBoss exploit used in the attack [19]. In addition, several of our recommendations, synthesized from a variety of sources, map directly to the Health Insurance Portability and Privacy Act (HIPAA) Security rule [20].

## Step 1 – Ensure Adequate System Protection by Correctly Installing and Configuring Computers and Networks

The computing infrastructure must be prepared for a ransomware attack by creating a regular backup process for the data. This backup should be made frequently (i.e., at least daily, and a continuous or real-time backup is ideal). Copies of these backups should be stored offline to ensure that ransomware has no access to them. In addition, organizations should maintain a “gold image” of system configurations (i.e., one that allows an organization to reset systems to the pre-attack state). One should also test the organization’s ability to restore these backups on a regular basis (e.g., quarterly for key data resources, yearly for less important aspects of the system).

Personnel in the organization responsible for maintaining all of the computers’ operating systems, application software, browsers and plug-ins, firmware, and anti-virus software should ensure that they are up-to-date with the latest patches. Before applying any patches, health IT professionals should thoroughly test them, along with the rest of the technical and application infrastructure, to ensure that the patches do not create new, unforeseen problems. Network engineers should also ensure that the organization’s firewall is properly configured (e.g., require passwords on Remote Desktop Protocol [RDP] ports), to prevent unauthorized people or programs from accessing mission-critical organizational resources. It may be necessary to segment the network by categorizing IT assets (e.g., desktops, servers, routers), data, and personnel into groups and restricting access to these groups using entry and exit traffic filtering. Finally, at the local device level, organizations should consider disabling USB (Universal Serial Bus) ports to prevent malicious software delivery [21].

In addition to these hardware and software-specific precautions, organizations should develop a “whitelist” of specified programs that are allowed to run, while blocking all others in order to prevent malicious executables from running. Furthermore, the organization should consider blocking email messages with potentially weaponized attachments ([note: this is not an exhaustive list] *.exe, *.zip, *.rar, *.7z, *.js, *.wsf, *.docm, *.xlsm, *.pptm, *.rtf, *.msi, *.bat, *.com, *.cmd, *.hta, *.scr, *.pif, *.reg, *.vbs, *.cpl, *.jar files), from suspicious or unknown sources (e.g., sitwithheprop.ru or xxxiooo.com) [22, 23].

Organizations should also consider restricting the ability of users to “write” (i.e., create and delete files), on shared drives of departmental or group shares. They should also consider limiting users’ ability to install and run software applications using the principle of “Least Privilege,” or minimize users’ access to only those systems and services required by their job. This may include restricting users’ administrative privileges on local desktops and laptops. For users who require administrative access, configure two accounts, one with administrative privileges that is used only when necessary, and one with restricted privileges (e.g., no ability to install new applications), that they use for routine activities, including reading email and browsing the Internet.

## Step 2 – Ensure More Reliable System Defense by Implementing User-Focused Strategies

Once all the computers and networks are installed and configured appropriately, the next line of defense is adequate training so that users correctly operate their devices and applications. Additionally, health IT professionals should review organization-wide electronic messages to ensure they conform to criteria for “legitimacy” below. Health care organizations do not have to develop their own training courses for either their end-users or health IT professionals; many commercially available courses exist [24].

IT professionals must help create messages such that users can easily recognize them as legitimate e-mails. Specifically, legitimate messages from one’s own institution (e.g., employer’s IT department), should not ask users to download and run file attachments or ask them to enter account or password information. In addition, these messages should have a recognizable telephone number that can be cross-referenced in the local directory to enable an out-of-band check, or a personal email address with a legitimate user name that can be cross-referenced in the local directory. All email and website links should display the complete internet address (URL) to build trust.

End-users should be instructed on how to approach unrecognized emails with links and attachments. An example of such an approach is as follows:

- **First Hover** – on the link with your mouse pointer to identify where the link is taking you.
- **Take a Second to Think** – Any link, or attachment that is not from your own organization, or a recognized friend, should not to be clicked. When in doubt, either call or email (in a separate email) your friend or the organization requesting information to confirm it is legitimate.
- **Only When Sure, Click**

In addition to making end-users aware about the risks and proper responses to fraudulent email messages with attachments, health IT professionals should conduct simulated phishing attacks by sending fake (but safe) email messages or links to websites that appear to be from legitimate sources [25, 26]. They should also increase their ability to respond to a successful ransomware attack by periodically conducting mock system recovery exercises (i.e., identify backups and test restore capabilities).

Although this might be the norm at some places, all health IT departments should configure their virus protection software to scan all software downloaded from the internet prior to allowing users to execute it. They should also conduct regular risk and business impact assessments to identify key applications and data based on importance to the business (e.g., Tier 0 – essential for business operations; Tier 1 – 1 hour downtime acceptable; Tier 2 – 1 day downtime acceptable; Tier 3 – 1 week downtime acceptable). This could help develop a plan to manage a ransomware attack. Finally, the organization should require 2-factor authentication (i.e., something you have – token or cellphone, coupled with something you know – password), for remote access to applications.

## Step 3 – Ensure Comprehensive System Monitoring of Suspicious Activities

All organizations should develop a network and user activity monitoring system that conducts surveillance for suspicious activities (e.g., similar to the anomaly detection algorithms that credit card companies use to identify stolen cards) [27], such as receipt of email messages from known fraudulent sources, executable email attachments, unexpected changes in key files on network-attached drives, unknown processes encrypting files, or significant increases in network traffic on unexpected ports.

The organization should also continuously monitor the external environment for new security incidents (i.e., zero-day exploits, an attack that takes advantage of a security vulnerability on the same day that the vulnerability becomes generally known) [28], and address gaps and deficiencies as they are identified.

## Step 4 – Respond, Recover, Investigate, and Learn from Ransomware Attacks

Often the first indication that a ransomware attack has occurred is an alarming message sent to the user’s desktop background, or a window opens to a ransomware program that the user cannot close which contains instructions on how to pay the ransom. In these cases, users should turn off the computer and report it to their IT support team immediately. The IT professionals should disconnect the infected computer(s) from the network and turn off wireless network functionality of the infected machine. If the attack is widespread, the IT department should shut down all network operations (i.e., both wired and wireless), to prevent the malware from spreading.

Once the immediate threat is contained, the IT department should contact their organization’s insurance provider, a computer forensics expert, and in the USA, the Federal Bureau of Investigation’s (FBI) Internet Crime Complaint Center (http://www.ic3.gov/default.aspx). In addition, the organization should consider using an organization-wide password reset after recovery (i.e., immediately require all users to reset their passwords).

Following any unexpected extended system downtimes, whether caused by ransomware or some other human or naturally occurring event, the organization should convene a multi-disciplinary investigation team consisting of key administrative and clinical stakeholders and Health IT professionals [29] to review the event and its management, identify potential root causes, and discuss future prevention or mitigating procedures [30]. The organization should also consider consulting with external experts in IT system reliability to review and report on recommendations for improvements in key system components, configurations, and policies and procedures [31].

## Conclusions

With the recent rapid adoption of EHRs, the threat of ransomware in health care facilities is greater than ever. Simply sending an email message to all employees reminding them not to click on suspicious links or attachments in email messages is no longer sufficient to prevent the emerging threat of cyber-crime in the current, fast-paced, clinical computing environment. We outline a socio-technical approach to address ransomware that involves four overarching steps that health care organizations can undertake to secure an EHR and the underlying computing infrastructure. Similar to approaches to address other complex socio-technical health IT challenges, the responsibility of preventing, mitigating, and recovering from these attacks is shared between health IT professionals and end-users.

## Figures and Tables

**Table 1:** An Eight Dimensional Socio-technical Approach for Preventing or Mitigating Ransomware Attacks. (Based on Sittig & Singh’s Eight Dimensional Socio-technical model) [32]

Socio-technical dimension	Recommendations for Health Care Organizations
Hardware/Software	Perform regular backups of your data. Be sure to back up frequently (continuous or realtime backup may be ideal), and store your backups offline Maintain a “gold image” of system configurations (i.e., allows an organization to reset systems to the pre-attack state) Test your backup’s restore function regularly (e.g., quarterly for key data resources, yearly for less important aspects of the system) Keep your operating system, application software, browsers and plug-ins, firmware, and anti-virus software up-to-date with the latest patches Make sure your firewall is properly configured (e.g., require passwords on Remote Desktop Protocol [RDP] ports) Segment your network by categorizing IT assets (e.g., desktops, servers, routers), data, and personnel into groups, and restricting access to these groups using entry and exit traffic filtering Consider disabling USB (Universal Serial Bus) ports to prevent malicious software delivery Following a successful attack, disconnect the infected computers from the network Turn off wireless network functionality of the infected machine If the attack is widespread, shut down all network operations to prevent the malware from spreading
Clinical Content	“Whitelist”, or allow only specified programs to run, while blocking all others, to prevent malicious executables from running Block email messages with attachments *.exe, *.zip, *.rar, *.7z, *.js, *.wsf, *.docm, *.xlsm, *.pptm, *.rtf, *.msi, *.bat, *.com, *.cmd, *.hta, *.scr, *.pif, *.reg, *.vbs, *.cpl, and *.jar from suspicious sources
User Interface	Legitimate Messages Should Have A Telephone Number Someone Can Call (I.E., Out Of Band Check), And A Personal Email Address Which Has A Legitimate User Name That People Can Check In Their Local Directory; Email And Website Links Should Display Complete Internet Address (Url) To Build Trust Often The First Indication That An Attack Has Occurred Is An Alarming Message Sent To The Desktop Background, Or A Window Opens To A Ransomware Program That You Cannot Close, With Instructions On How To Pay The Ransom; Users Should Turn Off The Computer And Report It To Their It Support Team Immediately
People	Do not follow unsolicited Web links in emails Users are trained on ransomware prevention strategies, including how to identify malicious e-mails (i.e., spam, phishing, and spear-phishing messages), and avoid clicking on potentially weaponized attachments (such as a *.exe, *.zip, *.rar, *.7z, *.js, *.wsf, *.docm, *.xlsm, *.pptm, *.rtf, *.msi, *.bat, *.com, *.cmd, *.hta, *.scr, *.pif, *.reg, *.vbs, *.cpl, *.jar files). Safe file attachment formats include *.jpg, *.png, *.pdf, *.docx, *.xlsx, and *.pptx Train users not to use USB flash drives unless the drives are obtained from a trusted source Restrict users’ administrative privileges on local desktops and laptops. For users who require administrative access, configure 2 accounts, one with administrative privileges that is used only when necessary, and one with more restrictive privileges that they use for routine activities, including reading email and browsing the Internet Restrict the ability of users to “write” (i.e., create and delete files), on shared drives of departmental or group shares
Workflow/communication	Scan all software downloaded from the internet prior to executing it Conduct simulated phishing attacks (i.e., fraudulent email messages or websites that appear to be from legitimate sources), to raise user’s awareness of the problem Conduct mock system recovery exercises (i.e., identify backups and test restore capabilities) Conduct regular risk assessments Require 2-factor authentication for remote access to applications Consider using organization-wide password reset (expiration) in response to a successful attack
Internal Policies, Procedures, and Environment	Based on risk and business impact assessments, identify applications and data based on importance to the business (e.g., Tier 0 – essential for business operations; Tier 1 – 1 hour downtime acceptable; Tier 2 – 1 day downtime acceptable; Tier 3 – 1 week downtime acceptable) – Develop a plan to manage a ransomware situation accordingly Restrict users’ ability to install and run software applications using the principle of “Least Privilege,” or minimize users’ access to only those systems and services required by their job IT security should be under the control of executives with extensive IT experience (e.g., CIO or Chief Information Security Officer) Consider blocking users’ access to personal email accounts (e.g., Gmail, Yahoo, Hotmail, iCloud) and web advertisements to avoid malvertising (i.e., insertion of malicious code into online advertisements to infect unsuspecting users)
External Rules and Regulations	Review all information security-related HIPAA requirements Contact your organization’s insurance provider, a computer forensics expert, and the FBI in the event of a successful attack
Measurement and Monitoring	Monitor network activity to identify suspicious activity Monitor the external environment for security incidents and address gaps and deficiencies as they are identified Following unexpected extended system downtime (e.g., ransomware), convene an investigation team consisting of key stakeholders and Health IT professionals to review the event and its management, identify potential root causes, and discuss future prevention or mitigating procedures
